# The effect of oceanic mesoscale eddies on the looping path of the Kuroshio intrusion in the Luzon Strait

**DOI:** 10.1038/s41598-020-57487-9

**Published:** 2020-01-20

**Authors:** Qian Yang, Hailong Liu, Pengfei Lin

**Affiliations:** 1 LASG, Institute of Atmospheric Physics, Chinese Academy of Sciences, Beijing, 100029 China; 2 College of Earth and Planetary Sciences, University of Chinese Academy of Sciences, Beijing, 100049 China

**Keywords:** Physical oceanography, Physical oceanography

## Abstract

In this study, the effects of oceanic mesoscale eddies on the looping path of the Kuroshio intrusion (KI) were symmetrically investigated by composite analysis using merged satellite data. We found that the mesoscale eddies propagating from the east have a significant impact on the looping path over a time scale of 30–60 days. Cyclonic eddies (CEs) enhance the looping path, but anticyclonic eddies decrease it. We also found that strong eddies do not have strong effects on the looping path. For instance, strong CEs induce the strong surface intrusion of the Kuroshio, but the looping currents are weak due to the presence of the strong Luzon Cold Eddy in the South China Sea, which tends to prevent loop formation. The complicated relationship between eddies and the path of the KI results in a nonsignificant correlation coefficient between the KI and eddy activities in the western Pacific.

## Introduction

The Kuroshio, the western boundary current of the North Pacific subtropical gyre, commonly penetrates into the South China Sea (SCS) when passing by the Luzon Strait (LS). In addition, the SCS is an important marginal sea in terms of regional weather and climate and may also affect the Western Pacific and Eastern Indian Warm Pool regions. The Kuroshio intrusion (KI) not only affects the properties of the northern SCS (e.g., stratification, circulation and mesoscale eddies) but also affects the mass, heat and salt budgets of the entire SCS basin^1–6^.

The Kuroshio may intrude into the SCS through the LS via different paths. Four or five different paths have been identified in earlier studies^7–9^. Nan *et al*.^10,11^ classified the KI paths into three major types based on the area-averaged geostrophic vorticity (GV) in the southwestern Taiwan: leaping, looping and leaking paths. Because an anticyclonic looping path usually forms when the Kuroshio is weak and easily affected by the eddies, we are mainly focused on the looping path in this study.

Many different dynamical mechanisms have been proposed to alter the paths of the KI. The path that the KI takes through the LS is primarily determined by the balance between the inertia and beta effects with some hysteresis effects^12^. The local topography in the LS^13,14^ and the incident angle and velocity of the Kuroshio^12,15^ are also important in modifying the paths of the KI by changing the inertia effects, the beta effect and the potential vorticity balance in this region. In addition, some studies have suggested that circulation^16^ and surface winds^17^ in the SCS may play important roles.

Recently, the effects of oceanic mesoscale eddies on the Kuroshio and KI have received substantial attention with the increasing horizontal resolution of both observational datasets and numerical models^11,16,18–23^. Cyclonic eddies (CEs) may reduce the Kuroshio transport by affecting the zonal gradient of the SLA^21^ or SSH^18^ or by the resulting upstream convergence and downstream divergence^22^, while the anticyclonic eddies (AEs) have the opposite effect. The enhanced/reduced transport in the Kuroshio may further change the KI pathway in the LS. Some westward propagating CEs may lead to enhanced looping currents^24^. However, the correlation between the paths of the KI and eddy activities in the western Pacific is not statistically significant^25^. The role of the mesoscale eddies from the Pacific on the KI path through the LS is still controversial.

**Figure 1:**
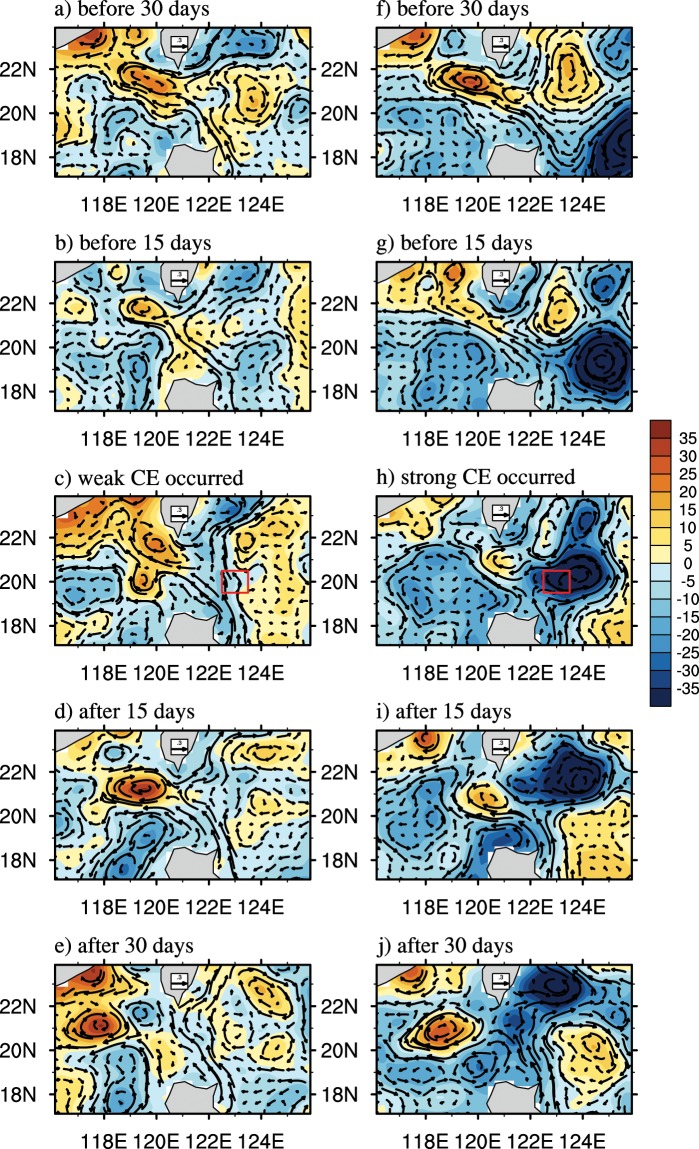
The evolution of SLA (shaded, cm) and the associated surface geostrophic current anomalies (vector, m/s), before and after the CE appears in the hotspot region (19.5°–20.5°N, 122.5°–123.5°E, red box), which can affect the looping path due to the interaction between the eddy and Kuroshio. (**a**–**e**) A weak CE case from December 27, 1999 to February 25, 2000. (**f**–**j**) A strong CE from December 22, 1994 to February 20, 1995.

The purpose of the present paper is to demonstrate the dominant impact of oceanic mesoscale eddies on the looping path of the KI using merged satellite data. The looping path accounts for approximately 15% of the KI and is mostly sensitive to the impacts of ocean mesoscale eddies compared with the other two paths (see Supplementary Fig. S1). This difference arises mainly because the Kuroshio is relatively weak and can be easily disturbed by eddies when a looping path occurs. We find that many CEs may propagate from the east to reduce the Kuroshio transport and then strengthen the looping path in the LS (see the two evolution examples in Fig. 1). However, a stronger CE may not lead to a stronger looping path magnitude, suggesting a nonlinear and complicated relationship (comparing Fig. 1c and h). By examining the evolution of the looping currents by using the Kuroshio Warm Eddy Index (KWI)^26^ every 5 days (figures not shown), which is defined to describe the anticyclonic eddy in the SCS formed by the Kuroshio loop (details in the Methods section), we find that the loop for a weak CE becomes stronger than that for a strong CE when the CE occurs. Here, we performed a composite analysis on the looping paths when eddies (both CEs and AEs) are present east of the LS and further investigate the magnitudes of the looping currents when an interaction occurs between the Kuroshio and stronger/weaker CEs or AEs. This paper represents the first study attempt to systematically clarify the connection between mesoscale eddies in the Pacific Ocean and the looping path in the LS. The results of this study will help us to gain a better understanding of the effects of the mesoscale process in the Pacific on the circulation and eddies of the LS as well as the effects on their interactions.

## Results

### The effects of eddies on the looping path

**Figure 2:**
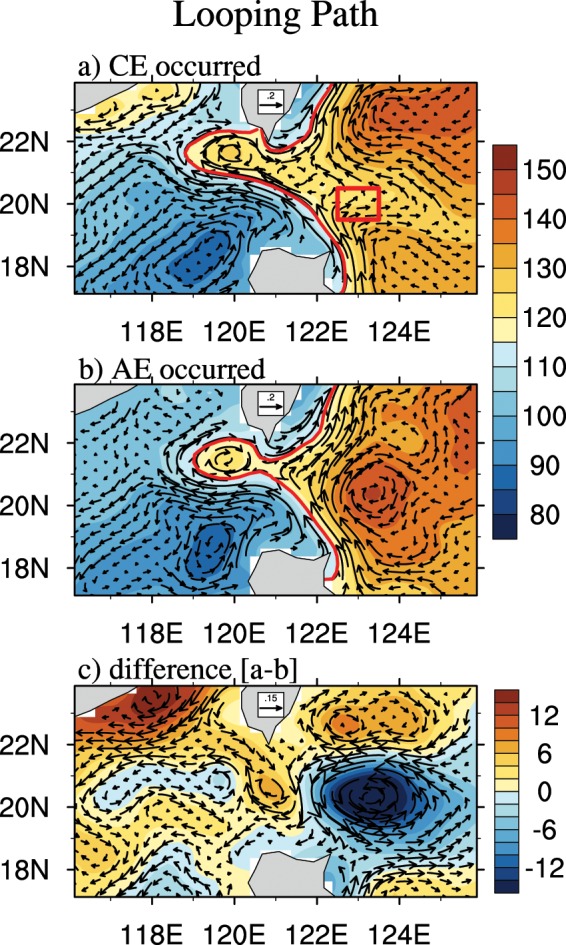
The composited ADT (shaded, cm) and associated surface geostrophic currents (vector, m/s) of the looping path for the occurrence of (**a**) CEs and (**b**) AEs in the hotspot region. The occurrence of eddies is defined by the red box (same as that shown in Fig. 1). The red curves represent the Kuroshio axis as determined by the zero contour of the GV. (**c**) The difference between (**a**,**b**).

In the present study, we performed a composite analysis to investigate the effects of an eddy on three different KI paths. According to previous work^23,27^ and the characteristics of eddies in our study, we select a key region (area in the box: 19.5°–20.5°N, 122.5°–123.5°E) as the place where eddies begin to interact with the Kuroshio, which means that the beginning of the interaction is defined as when an eddy can be detected in the key region. The details of the composite method can be found in the Methods section. Here, we use the strong looping path to perform the composite analysis to present our results more clearly. The composited absolute dynamic topography (ADT) and corresponding surface geostrophic currents with the CE and AE occurrences and their difference when the looping path occurs are shown in Fig. 2. To show the position of the eddies more clearly, we also compared the anomalous field (see Supplementary Fig. S2). The red curves represent the Kuroshio axis as determined by the zero contour of the GV. The comparison between AEs and CEs shows that the CEs force a stronger KI that moves in a more westward direction into the SCS than the KI that the AEs force (Fig. 2). We examined all CE and AE cases, and 17 (23) out of 24 (36) had a stronger (weaker) KI when the CEs (AEs) were present, reflecting a ratio of approximately 2/3. Therefore, we believe that we can obtain the same conclusion if we use all the cases in the composite.

Clearly, the CEs east of the LS block the upstream Kuroshio transport by reducing the ADT gradient across the Kuroshio and producing offshore currents (Fig. 2a). The AEs have the opposite effect (Fig. 2b). The weak (strong) Kuroshio and strong (weak) KI due to the presence of CEs (AEs) are more evident in Fig. 2c, as reflected by the southward anomalies of the surface current in the LS and the positive anomalies (6 cm) of ADT southwest of Taiwan at approximately 22°N. Notably, the eastward anomalous currents at the northern end of the LS seem to come from the Taiwan Strait; this finding might be attributed to the large biases of the satellite altimeter data in the shallow water region. Previous studies of observational and numerical results^28,29^ also noted that the reverse flow (southwestward) in winter with roughly a biweekly period might be caused by the atmospheric biweekly winter fronts. The leaping and leaking paths are also investigated but with only weak responses (see Supplementary Fig. S1).

**Figure 3:**
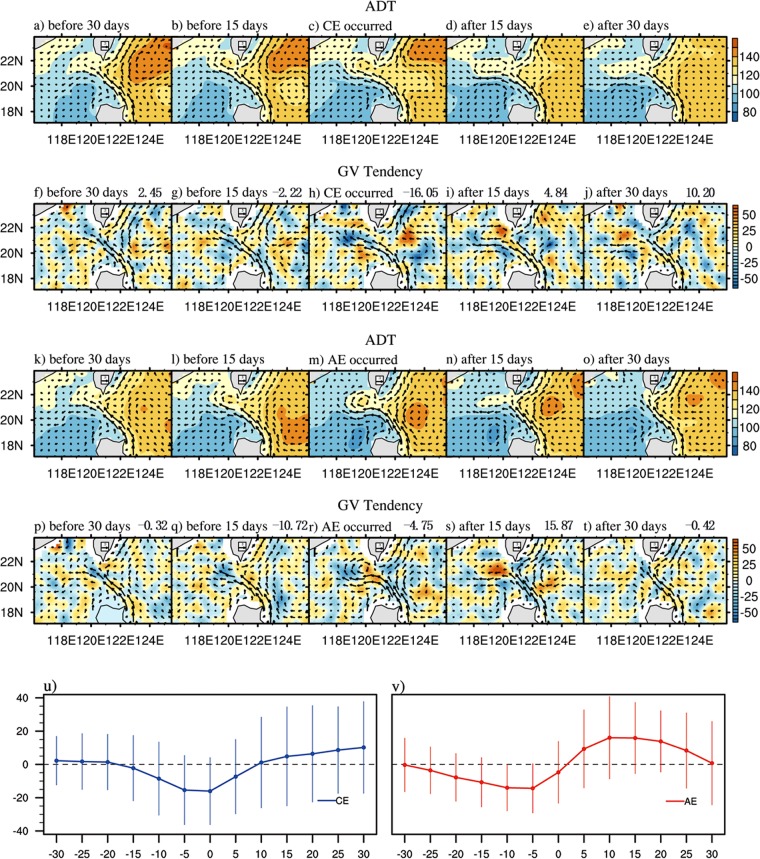
(**a**–**e**) The evolution of the composited ADT (shaded, cm) and the associated surface geostrophic currents (vectors, m/s) for the occurrence of the CEs. Every 15 days are shown. (**f**–**j**) The same as in (**a**–**e**) but for the GV tendency (×10^−13^ *s*^−2^) at the corresponding time. (**k**–**t**) The same as in (**a**–**j**) but for the AEs. The number in the upper right corner of each panel represents the area-averaged GV tendency within the loop (20°−22°N, 118.5°–120.5°E). The evolution of the composited GV tendency (s^−2^) for the occurrence of the (**u**) CEs and (**v**) AEs and their standard deviation. The x axis represents the days before and after the CEs (AEs) occurred in the key region for the first time.

To further study the impact of AEs and CEs on the KI, we also composited the ADT and surface currents both before and after CE/AE occurrence in the interaction area in Fig. 3a–e,k–o. Therefore, the propagation of the CEs/AEs and the development of the KI paths can be clearly observed. For example, CEs appeared west of 125°E before approximately 30 days. As the CE gradually moves westward, the KI develops from a leaping path into a looping path, and the ADT in the loop reaches its maximum when CEs arrive to the interaction area. Simultaneously, the anticyclonic GV in the loop strengthens. After 15 days, an AE gradually falls off in the loop with CEs moving northward, and the Kuroshio finally returns to the leaping state after 30 days, when the CEs move east of Taiwan. This finding indicates that when the Kuroshio is weak, the effect of the CEs on the intrusion is significant over a time scale of approximately 30–60 days. The development process is similar to the case analysis by Zhang *et al*.^30^ and Kuo *et al*.^23^. However, in addressing how the AEs interact with the Kuroshio (Fig. 3k–o), we find that the loop is much weaker and that the anticyclonic current in the southwestern Taiwan seems to fall off earlier.

We also analyzed the development of the GV tendency in the loop (20°–22°N, 118.5°–120.5°E). From the development of the GV tendency, we can determine the speed of the looping path development. With the westward movement of the CE, the increased GV trend decreases, as shown in Fig. 3f–j. The GV tendency reaches its local negative maximum when the CEs arrive at the interaction area; then, the decreasing trend begins to weaken, which corresponds to the AE in the loop beginning to drop along with the northward propagation and decay of the CEs east of the LS. However, as the AEs reach the key region east of the LS, the negative GV tendency increases gradually, indicating that the looping path reduces. A number of trial calculations with different areas of integration were computed, and the results were qualitatively consistent.

We also calculated the time series of the GV tendency every 5 days (Fig. 3u,v), and we found that the GV tendency due to AEs is approximately 5–10 days leading the CEs. The loop is strengthened (weakened) when the CEs (AEs) occur in the key region. This means that the strengthening or weakening of the loop due to eddies also correlate with the different timing. According to previous studies by Zhao and Luo^18^, the influence of the eddies on the mean current depends upon both the type of eddies and their relative positions. The upstream Kuroshio is weakened (enhanced) as the AE (CE) is still some distance away from it, whereas the upstream Kuroshio is enhanced (weakened) as the AE (CE) moves near or within the mean current. Therefore, the KI is enhanced (weakened) when the AE (CE) is far from the Kuroshio but weakened (enhanced) when the AE (CE) interacts with the Kuroshio.

To further understand the differences between the AE and CE situations, we computed the changes in the GV using Eq. (5), as described in the Methods section. An obvious weakening in the GV is shown in southwestern Taiwan for both the CEs and AEs (see Supplementary Fig. S3a,f). The GV budget terms were averaged for the area of looping currents for two situations, which shows that the GV in the loop when the CEs occur decreases much more than it does in the AE situations, which is primarily due to the advection effect and the *β* term (Table 1). That is, when CEs occur, more negative GV transports into the looping currents, and then the Kuroshio tends to intrude in a way that is stronger and more westward. To test how well the vorticity can be quantified by the coarse spatial and temporal resolutions of the Archiving, Validation and Interpretation of Satellite Oceanographic (AVISO) data, we also calculated the budget in a quasiglobal eddy-resolving (10 km) model, the LASG/IAP Climate system Ocean Model (LICOM)^31,32^. The experiment is a standard Coordinated Ocean-ice Reference Experiments phase II (CORE-II) type of experiment^33^, but it is not coupled with the sea-ice model. The relationships among these terms are qualitatively the same as the observations, but these terms have slightly larger values. Therefore, it is suitable to use the AVISO data to quantify the GV budget.

**Table 1:** The regional mean major terms of the GV budget (Eq. (1)) within the loop (20°–22°N, 118.5°–120.5°E) for CEs and AEs at −10 days, −5 days, 0 days, 5 days and 10 days (unit: ×10^−13^ *s*^−2^).

		Tendency	Advection	Stretching (GV)	Stretching (f)	Beta*v
−10 days	CE	−8.55	−2.59	0.38	17.01	−20.51
	AE	−14.03	7.24	−0.05	14.56	−17.38
−5 days	CE	−15.41	−2.33	0.25	18.47	−22.35
	AE	−14.35	1.08	−0.17	15.04	−17.93
0 days	CE	−16.05	−8.01	0.02	16.43	−19.95
	AE	−4.75	7.03	−0.15	11.12	−13.22
5 days	CE	−7.34	−8.23	0.06	12.27	−14.82
	AE	9.31	19.20	0.32	5.36	−6.16
10 days	CE	1.15	−9.40	0.17	7.44	−8.89
	AE	16.04	20.20	0.94	1.79	−1.79

The GV budget at −10 days, −5 days, 5 days and 10 days are also computed to show the changes in the GV budget during the interaction between the Kuroshio and eddies (Table 1). For the CEs, the decrease in the advection term leads to a decrease in the GV tendency before the minimum, and subsequently, the *β* term that leads to an increase in the GV tendency. For the AEs, the situation is the same as that for the CEs, in which the decrease in the advection term leads to a decrease in the GV tendency before the minimum, and subsequently, both the advection and the *β* terms lead to an increase in the GV tendency.

### Weak/strong cyclonic eddies and the looping path

**Figure 4:**
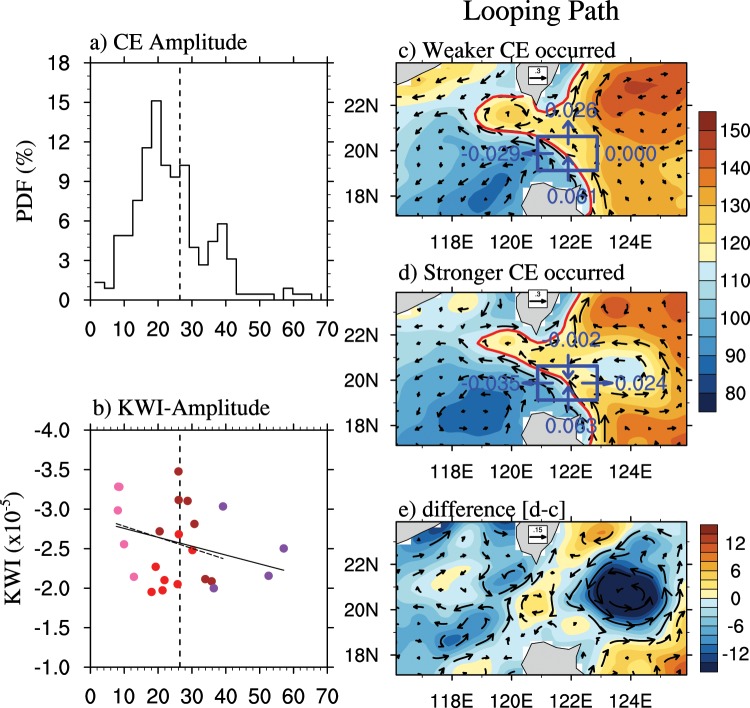
(**a**) The probability density function of the CE amplitude in the hotspot region (red box in Fig. 1). (**b**) The scatter plot between the CE amplitudes (x axis, cm) and the KWI (y axis, ×10^5^ *s*^−1^). Different eddy radii are represented by different colors. (**c**) The composited ADT (shade, cm) and the associated surface geostrophic currents (vectors, m/s) for the occurrence of weak CEs. The red curves represent the Kuroshio axis as determined by the zero contour of the GV. The transport of the upper 1 m depth across the blue box (19°–21°N, 121°–123°E) is shown (unit: Sv). The directions are also provided as vectors. (**e**) The difference between (**d**,**c**).

As shown in Fig. 1, the CEs arriving at the key region have different intensities, but the relationship between the CE intensity and the looping current intensity is not linear. We further analyzed the roles of different intensity eddies on the looping path. Here, we focus on the CEs. The intensities of eddies are measured using the amplitude, which is the difference in the sea level anomaly (SLA) between the center and edge of the eddies, and we do not consider the state of the eddies. Figure 4a shows the probability density function of all the CEs. The amplitude of the weakest CE is only 3 cm, while that of the strongest CE reaches 68 cm, and the mean amplitude is 20 cm. Therefore, we chose an amplitude of 26.45 cm (dashed line), which was the mode value of the CE amplitude during the looping path as the criterion for strong and weak eddies. The strong and weak eddies lasted for 45 days and 75 days, respectively. Other criteria, such as the mean value and the median value, also have been tested, and the results are not very different.

The composited ADT and corresponding surface geostrophic currents for the weak and strong CEs and their differences are shown in Fig. 4c–e. Interestingly, the looping paths for the weak CEs reach slightly more to the west (119.1°E vs. 118.7°E), and the corresponding loop is much stronger than that of the strong CEs. The volume budget is computed where the Kuroshio and CE interact. The range in the blue box is 19°–21°N, 121°–123°E. Here, we assume that the depth of the surface layer is 1 m. Clearly, the strong CEs cause a considerable Kuroshio transport into the LS. The Kuroshio transport east of the LS is convergent in the meridional direction and divergent in the zonal direction. When strong CEs interact with the Kuroshio, the convergence is greatly enhanced, and the transport at the northern boundary changes from 0.026 Sv northward to 0.002 Sv southward. Thus, the Kuroshio transport into the LS is enhanced due to the increasing zonal divergence. However, the volume budget is not balanced because the vertical transport cannot be measured due to the observational data limits. The currents around the LS are trapped at the surface, so these currents do not have much of an impact on our conclusions.

However, the enhanced Kuroshio transport into the SCS does not lead to a strong looping path. In comparing the circulation in the SCS for the weak and strong CEs, we found that there is a strong cyclonic eddy, which is usually called the Luzon Cold Eddy (LCE, e.g., Shaw *et al*.^34^), located in the south of the looping current during the strong CE. Therefore, the strong KI does not flow northwestward to form the looping path; instead, it flows southwestward to enhance the LCE. In other words, there are other factors within the SCS that can affect looping path formation. In addition, we calculated the GV tendency for the occurrence of the LCEs without considering the eddies east of the LS. The negative trend caused by the LCEs is much smaller, so we speculated that the impact of the LCE on the looping path is not as large as that of the CEs east of the LS. Additionally, we counted the amplitude of the CEs east of the LS when the LCE occurred to show that a stronger LCE does not always accompany stronger CEs (weaker AEs) east of the LS.

We also evaluated the relationship between the LCE and the looping path in LICOM^31,32^ (figure not shown). We composited the looping path when there is and is not a LCE present and found that there is no significant change in the looping path, indicating that the relationship between the LCE and the looping path may not be stable and that the LCE may not be the dominant influence on the looping path. This finding also indicates that the relationship between the eddies east of the LS and KI may dominate the entire relationship.

Furthermore, we drew a scatter plot of the amplitudes of the CEs and the KWI (Fig. 4b). The regression shows that the strong CEs will lead to weak looping paths, but the slope is small (solid line). If we remove two strong CEs (occurring on January 21 and 26, 1995), the regression line does not change very much (dashed line). This is because there are at least three issues that may influence the loop, as mentioned above, including the Kuroshio eddies east of the LS and LCEs. Moreover, there are fewer examples in the observational data. We tested the relationship between strong/weak CEs and the loop using an eddy-resolving model and obtained the same results. This finding can explain the low correlation between the KI and mesoscale signals in the Pacific, as discussed by Nan *et al*.^25^.

We also investigated the effect of the AE intensity (see Supplementary Fig. S4). The result is the same as that for the CEs in that the weak AEs will reduce the loop much more significantly than the strong AEs. The strong LCE is still an important factor that affects the KI.

## Conclusions

In the present study, the effects of mesoscale eddies, both CEs and AEs, on the looping path of the KI were investigated through composite analysis using satellite observations. We found that the mesoscale eddies propagating from the east have a significant impact on the looping path when the upstream transport of the Kuroshio is weak, which we noted over a time scale of 30–60 days. The CEs will enhance the looping path, and the AEs will decrease the looping path. However, eddies seem to have relatively little effect on the leaping and leaking paths due to the strong Kuroshio. This result is consistent with the findings of Yuan and Wang^19^, who found that the KI is not always affected by a mesoscale eddy, and the Kuroshio is only sensitive when it is near the critical states of hysteresis.

The intensity of the eddies on the looping current magnitudes was further analyzed. We found that a smaller magnitude of the looping path corresponds to stronger CEs or weaker AEs. This finding is surprising and different from what we believed at the beginning of this study. The strong CEs do induce the strong intrusion of the Kuroshio in our volume budget analysis, but the looping currents are weak due to the unfavorable eddies or circulations in the SCS. Here, the strong LCE prevents loop formation. A recent study by He *et al*.^35^ showed that the interaction between the LCE and KI is important for the development and maintenance of the LCE, and inversely, the LCE will influence the state of the KI. Zhong *et al*.^16^ has also mentioned that the circulation in the SCS may affect the KI in addition to the eddies. The complicated relationship between the eddies and the KI path results in a nonsignificant correlation coefficient between the KI and eddy activities in the western Pacific, as shown by Nan *et al*.^25^. However, these relationships among the KI, LCE and eddies east of Luzon are worth further investigation.

## Methods

### The composite method

In the present study, we performed a composite analysis to investigate the effects of the eddy on three different KI paths. First, the KI was classified into three types, namely, the leaping, looping and leaking paths, using the method of Huang *et al*.^26^ over the region 20°–22°N, 119°–121°E. Two indices, collectively called the Double Index (DI), are defined by the KWI and the Kuroshio Cold Eddy Index (KCI) (see Supplementary Fig. S5). The criterion for the looping (leaping) path is the KWI (KCI) below (above) its standard deviation, and the value of the area-averaged GV is also a measurement of the intensity of the looping path. The details of the current spatial patterns (see Supplementary Fig. S6) can be found in the Supplementary Material.

Second, we identified all of the eddies that interacted with the Kuroshio east of the LS in the region 19.5°–20.5°N, 122.5°–123.5°E (red box in Fig. 2a), where the eddies and Kuroshio frequently interact during the 1993–2016 period according to Cheng *et al*.^36^. Other interactive areas have already been tested, but the composite results are quantitatively similar among these areas. The eddy detection and tracking method used in this study is based on that of Lin *et al*.^37^. Following our previous studies^38^, we focused on eddies only with lifetimes longer than 5 weeks, amplitudes larger than 3 cm and water depths greater than 200 m. Tables S1 and S2 show the CE and AE properties. We found that the total staying time for the CEs (AEs) is 120 (185) days, as shown in the box, over the 1993–2016 period when the looping paths occur. The dates of the CEs and AEs that appear in the red box are also marked by blue and pink dashed lines in Fig. S4. The average amplitude and radius of the CEs (AEs) are 23.16 cm (18.73 cm) and 143.55 km (135.53 km), respectively.

Finally, we created a composite based on both the KWI index and the occurrence of eddies in the key region. The composition of the ADT and the corresponding geostrophic current according to the different polarity and amplitude values of the eddies are evaluated and compared.

### Double index

The DI, which was defined by Huang *et al*.^26^, is used to derive the types of KIs. The DI is defined as the areal integral of the positive and negative geostrophic vorticities over a region (20°–22°N, 119°–121°E), respectively, and the two indices are called the KCI and KWI. The formulas are given below.

1 $${\rm{KWI}}=\oiint {\rm{sign}}\,(\,-\,\text{GV})\text{GVdA}$$

2 $${\rm{KCI}}=\oiint \text{sign}(\text{GV})\text{GVdA}$$

where the sign function and GV are defined as follows:

3 $${\rm{sign}}({\rm{x}})=\{\begin{array}{ll}1, & x\ge 0\\ 0, & x < 0\end{array}$$

4 $${\rm{GV}}=\frac{\partial v}{\partial x}-\frac{\partial u}{\partial y}$$

We use the standard deviations of the KWI and KCI as thresholds to obtain the three types of KI paths. When the result of the calculation is smaller than the standard deviation of the KWI, the event is defined as the looping path. When the result is greater than the standard deviation of the KCI, the event is defined as the leaping path. The remaining event reflects the leaking path. However, if the KWI and the KCI both satisfy their criteria, we will normalize the two indices. When the absolute value of the normalized deviation of KWI (KCI) is larger, the event reflects the looping (leaping) path. The details of the three paths are briefly described below. The looping path is characterized by a strong anticyclonic circulation southwest of Taiwan Island^10,11,26^, and the Kuroshio water enters the SCS in the middle and outflows in the northern part of the LS. This action forms a “Kuroshio Current Loop”^39^ southwest of Taiwan Island. AEs can be shed from the loop^7,23,30^. Thus, the GV in the loop is negative. When there is a strong cyclonic circulation in the area west of the LS and the Kuroshio flows directly northward and passes by the LS, we say the Kuroshio has a leaping path at this time. The rest of the situations reflects the leaking path.

In this paper, we analyze only the cases that have strong loops. Specifically, we chose these loops by taking the standard deviation of the KWI after the looping paths had already been diagnosed. We have also investigated the results of all the looping paths, which are similar to those of the stronger cases.

### Eddy detection and tracking method

We use the method similar to the Winding Angle (WA) method^40^ in this paper to detect eddies, and this method has been widely applied to studies of the SCS^38,41^, the Atlantic Ocean^40^ and broader global oceans^42^. The first step is to identify the possible CE (or AE) centers by searching for local SLA minima (or maxima) in a moving 1° × 1° grid. Next, the algorithm searches for closed contours with an increment (or decrement) of 1 mm for each possible CE (or AE) center. Then, the outermost closed SLA contour that encloses only one extreme center is considered the edge of the eddy. In addition, the eddy tracking method is based on the geometrical distance from one eddy center to another^43,44^. The MATLAB code for this method was obtained from Lin *et al*.^37^ and slightly modified. The CEs/AEs that occurred in the looping path are labeled in the KWI and KCI with dashed lines in Fig. S4.

### Surface geostrophic vorticity budget

To further understand the interaction between the eddy and the Kuroshio, the surface GV was determined following the work of Kuo *et al*.^23^. The GV budget equation can be written as follows:

5 $$\frac{\partial \zeta }{\partial t}=-(u\frac{\partial \zeta }{\partial x}+v\frac{\partial \zeta }{\partial y})-\zeta (\frac{\partial u}{\partial x}+\frac{\partial v}{\partial y})-f(\frac{\partial u}{\partial x}+\frac{\partial v}{\partial y})-\beta v-B$$

where $\zeta$, *f*, *β* and *B* are the relative vorticity, Coriolis parameter, meridional change in f and the rate of change in $\zeta$ due to the friction and wind stress, respectively, which cannot be explicitly diagnosed as a certain variable. Thus, the trend in the relative vorticity can be balanced by the advection of the relative vorticity, the stretching term of the relative vorticity, the stretching term of the planetary vorticity, the beta term that represents the meridional advection of the planetary vorticity and other terms denoted by B.

### Observation data

The ADT and SLA data used in this paper are derived from merged satellite altimeter products from the AVISO dataset, http://www.aviso.oceanobs.com) during the 1993–2016 period. The spatial resolution of the data is 0.25° × 0.25°, and the temporal interval is 5 days, with averaging from the original daily products. The surface geostrophic currents are obtained through the geostrophic relation.

## Supplementary information

Supplementary Information
