# Supplementary material for: The effect of oceanic mesoscale eddies on the looping path of the Kuroshio intrusion in the Luzon Strait
Source: Sci Rep. 2020 Jan 20;10:636. doi: 10.1038/s41598-020-57487-9 (PMC6971238; doi:10.1038/s41598-020-57487-9)
Supplement: Supplementary file 1 — Supplementary Information [file 41598_2020_57487_MOESM1_ESM.docx]

**The effect of oceanic mesoscale eddies on the looping path of the Kuroshio intrusion in the Luzon Strait**

Qian Yang^1,2,^ Hailong Liu^1,2*^ and Pengfei Lin^1,2^

^1^ LASG, Institute of Atmospheric Physics, Chinese Academy of Sciences, Beijing 100029, China

^2^ College of Earth and Planetary Sciences, University of Chinese Academy of Sciences, Beijing 100049, China

* Corresponding author: Hailong Liu ([lhl@lasg.iap.ac.cn](mailto:lhl@lasg.iap.ac.cn))

**Contents of this file**

Figures S1 to S6

Table S1-S2

**Figure S1** The composition of the ADT (shaded, cm) and the associated surface geostrophic currents (vector, m/s) when a) CEs and b) AEs occurred in the eastern Luzon Strait, and c) is the difference between a) and b) during the leaping path. d)-f) is the same as a)-c), but for the leaking path. The red box (19.5°-20.5°N, 122.5°-123.5°E) denotes the eddy and Kuroshio interacting region and the key region where the eddies are computed. The red curves represent the Kuroshio axis as determined by the zero contour of the geostrophic vorticity (GV).


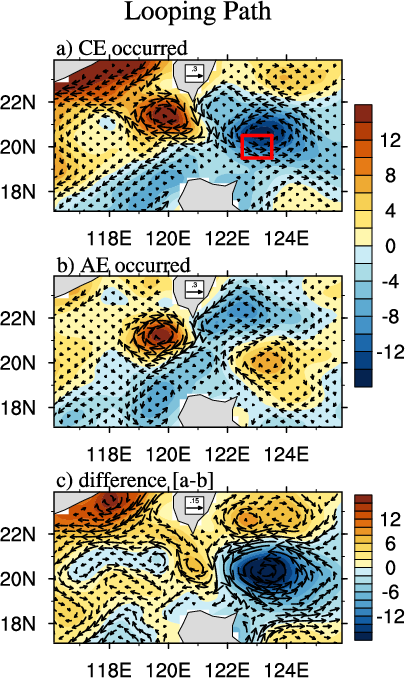


**Figure S2** The composited SLA (shaded, cm) and the anomaly of the surface geostrophic currents (vector, m/s) for the looping path for the occurrence of (a) CEs and (b) AEs in the key region. The occurrence of eddies is defined by the red box (same as that shown in Fig. R1). The red curves represent the Kuroshio axis as determined by the zero contour of the GV. (c) is the difference between (a) and (b).

**Figure S3** The composited major terms of the vorticity equation (×10^-13^ *s*^-2^) and the associated surface geostrophic currents (m/s) when a)-e) CEs and f)-j) AEs occurred in the eastern Luzon Strait.

**Figure S4** The same as Figure 4, but for AEs.

**Figure S5** The Kuroshio Warm eddy Index (KWI) and Kuroshio Cold eddy Index (KCI) as calculated using the method by *Huang et al.* [2016]. The indices are defined as the area-mean negative and positive geostrophic vorticity over 20°-22°N, 119°-121°E. The red points indicate when the looping paths occur, and pink and blue lines indicate when the AEs and CEs occur.

**Figure S6** The composite of the ADT (shaded, cm) and the associated surface geostrophic currents for three typical Kuroshio intrusion paths, namely the looping, leaping and leaking paths, as identified using the Double Index.

**Table S1** The occurrence date and properties for CEs over the eddy and Kuroshio interacting region (19.5°-20.5°N, 122.5°-123.5°E).

| Number | Date | Radius (km) | Amplitude (cm) | Duration (day) | Lifetime (day) | Generation Location | KWI (×10^5^ *s*^-1^) |
| --- | --- | --- | --- | --- | --- | --- | --- |
| 1 | 19950121 | 211.67 | 52.64 | 10 | 90 | 18.125N, 128.625E | -2.68 |
| 2 | 19950126 | 237.64 | 57.11 |  | 90 | 18.125N, 128.625E | -2.48 |
| 3 | 19981212 | 134.72 | 18.07 | 20 | 55 | 19.975N, 123.125E | -1.95 |
| 4 | 19981217 | 132.39 | 19.29 |  | 55 | 19.975N, 123.125E | -2.27 |
| 5 | 19981222 | 150.73 | 26.12 |  | 55 | 19.975N, 123.125E | -2.68 |
| 6 | 19981227 | 147.80 | 30.15 |  | 55 | 19.975N, 123.125E | -2.48 |
| 7 | 20000126 | 92.31 | 8.12 | 20 | 40 | 20.625N, 123.875E | -2.98 |
| 8 | 20000131 | 92.78 | 8.57 |  | 40 | 20.625N, 123.875E | -3.28 |
| 9 | 20000205 | 80.74 | 8.22 |  | 40 | 20.625N, 123.875E | -3.28 |
| 10 | 20000210 | 79.31 | 7.65 |  | 40 | 20.625N, 123.875E | -3.16 |
| 11 | 20010322 | 148.36 | 21.89 | 5 | 40 | 19.875N, 124.125E | -2.10 |
| 12 | 20030903 | 191.29 | 33.95 | 5 | 50 | 19.875N, 124.375E | -2.11 |
| 13 | 20041127 | 82.16 | 9.95 | 5 | 60 | 19.375N, 126.625E | -2.56 |
| 14 | 20050215 | 219.49 | 36.45 | 5 | 70 | 19.375N, 127.375E | -2.00 |
| 15 | 20060215 | 191.06 | 35.81 | 5 | 55 | 20.125N, 126.375E | -2.09 |
| 16 | 20071122 | 146.08 | 25.77 | 5 | 40 | 19.375N, 123.375E | -2.05 |
| 17 | 20091013 | 95.99 | 12.85 | 5 | 20 | 180625N, 124.625E | -2.14 |
| 18 | 20091107 | 144.95 | 21.31 | 5 | 40 | 20.875N, 122.125E | -1.97 |
| 19 | 20091222 | 233.61 | 39.17 | 5 | 135 | 18.875N, 128.625E | -3.03 |
| 20 | 20111207 | 191.57 | 26.07 | 5 | 60 | 20.625N, 123.125E | -3.12 |
| 21 | 20121107 | 161.42 | 20.45 | 15 | 100 | 20.875N, 125.125E | -2.72 |
| 22 | 20121112 | 190.53 | 28.72 |  | 100 | 20.875N, 125.125E | -3.10 |
| 23 | 20121117 | 188.18 | 20.73 |  | 100 | 20.875N, 125.125E | -2.81 |
| 24 | 20161227 | 161.30 | 26.02 | 5 | 40 | 21.625N, 125.625E | -3.48 |
| Mean | - | 154.42 | 25.21 | 8 | 62.5 | - | -2.59 |

**Table S2** The same as S1, but for AEs.

| Number | Date | Radius | Amplitude | Duration | Lifetime | Location | KWI |
| --- | --- | --- | --- | --- | --- | --- | --- |
| 1 | 19930101 | 106.26 | 10.99 | 10 | 45 | 19.875N, 122.625E | -3.35 |
| 2 | 19930106 | 134.82 | 15.10 |  | 45 | 19.875N, 122.625E | -3.65 |
| 3 | 19960302 | 123.30 | 24.70 | 15 | 90 | 22.125N, 126.875E | -3.62 |
| 4 | 19960307 | 145.89 | 21.13 |  | 90 | 22.125N, 126.875E | -4.22 |
| 5 | 19930312 | 107.70 | 23.19 |  | 90 | 22.125N, 126.875E | -2.51 |
| 6 | 19991212 | 188.38 | 25.61 | 5 | 45 | 20.125N, 124.875E | -3.16 |
| 7 | 20000210 | 107.56 | 12.17 | 5 | 35 | 20.625N, 124.625E | -1.94 |
| 8 | 20010220 | 82.16 | 15.60 | 35 | 60 | 19.875N, 123.125E | -2.74 |
| 9 | 20010225 | 82.13 | 17.31 |  | 60 | 19.875N, 123.125E | -2.93 |
| 10 | 20010302 | 80.84 | 13.77 |  | 60 | 19.875N, 123.125E | -2.34 |
| 11 | 20010307 | 148.65 | 14.78 |  | 60 | 19.875N, 123.125E | -2.03 |
| 12 | 20010312 | 134.72 | 11.85 |  | 60 | 19.875N, 123.125E | -1.85 |
| 13 | 20010317 | 109.49 | 15.68 |  | 60 | 19.875N, 123.125E | -2.10 |
| 14 | 20010322 | 107.86 | 17.14 |  | 60 | 19.875N, 123.125E | -1.91 |
| 15 | 20010501 | 136.63 | 17.26 | 5 | 35 | 20.125N, 124.125E | -2.50 |
| 16 | 20020809 | 136.79 | 11.65 | 5 | 60 | 21.625N, 125.125E | -2.15 |
| 17 | 20021112 | 164.00 | 19.46 | 10 | 45 | 21.375N, 124.125E | -2.43 |
| 18 | 20021117 | 177.89 | 25.17 |  | 45 | 21.375N, 124.125E | -2.52 |
| 19 | 20040317 | 107.95 | 11.56 | 5 | 40 | 19.625N, 125.375E | -3.24 |
| 20 | 20040705 | 164.18 | 39.07 | 5 | 50 | 20.375N, 125.875E | -2.97 |
| 21 | 20040809 | 237.87 | 62.46 | 5 | 50 | 20.375N, 124.875E | -2.41 |
| 22 | 20041207 | 132.60 | 18.69 | 15 | 45 | 19.875N, 126.875E | -1.83 |
| 23 | 20041212 | 107.86 | 15.76 |  | 45 | 19.875N, 126.875E | -1.76 |
| 24 | 20041217 | 79.13 | 8.76 |  | 45 | 19.875N, 126.875E | -1.82 |
| 25 | 20060406 | 123.50 | 13.34 | 10 | 55 | 19.625N, 122.375E | -1.69 |
| 26 | 20060411 | 123.34 | 12.66 |  | 55 | 19.625N, 122.375E | -1.96 |
| 27 | 20060426 | 109.53 | 17.00 | 5 | 55 | 19.625N, 122.375E | -2.09 |
| 28 | 20080101 | 148.36 | 16.58 | 5 | 45 | 19.125N, 126.125E | -2.62 |
| 29 | 20101102 | 229.10 | 32.18 | 5 | 35 | 20.375N, 125.125E | -2.74 |
| 30 | 20131107 | 159.27 | 25.48 | 35 | 75 | 19.875N, 124.625E | -3.03 |
| 31 | 20131112 | 161.42 | 25.02 |  | 75 | 19.875N, 124.625E | -2.98 |
| 32 | 20131117 | 163.93 | 19.73 |  | 75 | 19.875N, 124.625E | -2.67 |
| 33 | 20131122 | 211.94 | 25.30 |  | 75 | 19.875N, 124.625E | -3.03 |
| 34 | 20131127 | 225.48 | 23.27 |  | 75 | 19.875N, 124.625E | -2.98 |
| 35 | 20131202 | 188.47 | 24.13 |  | 75 | 19.875N, 124.625E | -2.67 |
| 36 | 20131207 | 187.81 | 25.69 |  | 75 | 19.875N, 124.625E | -1.94 |
| 37 | 20151227 | 148.25 | 24.20 | 5 | 35 | 19.875N, 123.125E | -3.05 |
| Mean | - | 142.84 | 20.36 | 10.28 | 57.43 | - | -2.55 |
